# Whole-Cell Multiparameter Assay for Ricin and Abrin Activity-Based Digital Holographic Microscopy

**DOI:** 10.3390/toxins11030174

**Published:** 2019-03-22

**Authors:** Efi Makdasi, Orly Laskar, Elad Milrot, Ofir Schuster, Shlomo Shmaya, Shmuel Yitzhaki

**Affiliations:** The Department of Infectious Diseases, Israel Institute for Biological Research, Ness-Ziona 74100, Israel; efim@iibr.gov.il (E.M.); orlyl@iibr.gov.il (O.L.); eladm@iibr.gov.il (E.M.); ofirsc@iibr.gov.il (O.S.); shlomosh@iibr.gov.il (S.S.)

**Keywords:** ricin, abrin, digital holographic microscopy, morphology, intoxication, holomonitor

## Abstract

Ricin and abrin are ribosome-inactivating proteins leading to inhibition of protein synthesis and cell death. These toxins are considered some of the most potent and lethal toxins against which there is no available antidote. Digital holographic microscopy (DHM) is a time-lapse, label-free, and noninvasive imaging technique that can provide phase information on morphological features of cells. In this study, we employed DHM to evaluate the morphological changes of cell lines during ricin and abrin intoxication. We showed that the effect of these toxins is characterized by a decrease in cell confluence and changes in morphological parameters such as cell area, perimeter, irregularity, and roughness. In addition, changes in optical parameters such as phase-shift, optical thickness, and effective-calculated volume were observed. These effects were completely inhibited by specific neutralizing antibodies. An enhanced intoxication effect was observed for preadherent compared to adherent cells, as was detected in early morphology changes and confirmed by annexin V/propidium iodide (PI) apoptosis assay. Detection of the dynamic changes in cell morphology at initial stages of cell intoxication by DHM emphasizes the highly sensitive and rapid nature of this method, allowing the early detection of active toxins.

## 1. Introduction

Ribosome inactivating proteins (RIPs) irreversibly damage ribosomes [1], leading to inhibition of protein synthesis and cell death [2]. Ricin and abrin—two well-known plant-derived toxins—are RIPs with similar structures, comprised of two glycoprotein chains (A and B) of equal sizes (~30 kDa) joined by a disulfide bond. The B-chain binds to galactose residues present on various cell surface glycoproteins and glycolipids, triggering endocytosis of the toxin. The A-chain exhibits a ribonucleic acid (RNA) N-glycosidase activity which depurinates a specific adenine residue located near the 3′ terminus of the 28S ribosomal RNA. This site-specific depurination event prevents binding of elongation factor 2 to the ribosome, thereby causing translational arrest [3,4]. Ricin and abrin are isolated from the seeds of castor bean plant *Ricinus communis* and *Abrus precatorius*, respectively. The ease and low cost of isolation make these proteins potent biothreat agents.

Currently, there are two main approaches for assessing ricin activity in noncell-based assays: measuring protein synthesis inhibition [5,6,7,8] and monitoring ribosomal 28S RNA depurination [9,10,11,12,13,14,15]. Although highly sensitive, these approaches focus on measuring the catalytic activity of ricin subunit A. Yet, to accurately assess the full activity of ricin toxicity, a cell-based assay should be employed. Current reported cell based assays for ricin toxicity measure cell survival (usually within 48 to 72 h postexposure) [16,17] or measure intracellular marker protein levels within 3–24 h postintoxication [6,18,19,20].

Cell death induction by apoptosis, necrosis, and autophagy is characterized by a distinct set of temporal morphological, biochemical, and gene expression features [21,22]. These specific morphological features, in particular changes in cell volume, accompany cell death processes. Thus, they are often used to differentiate between different cell death pathways—for example, loss of cell volume or shrinkage are morphological characteristics of apoptosis, while initial cell swelling is a characteristic of necrosis [22]. Several studies have shown that the cell death resulting from ricin and abrin intoxication is apoptotic in nature, induced by activating *caspase-3*. This has been demonstrated in several cancer cell lines as well as mammalian lymphoid tissues [23,24].

There are several commercial in vitro cell-based assays for assessing cell viability by the loss of membrane integrity. Such assays include nuclear staining of nonviable cells by trypan blue or propidium iodide dying [25,26] or measuring lactate dehydrogenase in the surrounding medium [27]. These assays are more pronounced and accurate at the late stages of cell death, but are destructive to the sample, require specific reagents and several hours for completion and are retrospective in their analysis. Time-lapse experiments for living cells using fluorescent microscopy are challenging to implement routinely and require substantial resources [28]. While signal specificity obtained from fluorescent microscopy is considered a major advantage, it suffers from some intrinsic limitations: requirement of exogenous labels, invasive procedures, appropriate reagents, and time-consuming image focusing process prior to image acquisition and analysis.

Digital holographic microscopy (DHM) is a time-lapse, label-free, and noninvasive imaging technique that can provide both quantitative and qualitative phase information on morphological features such as cellular area, shape, thickness, volume and confluence [29,30,31,32]. This method does not require any media changes or addition of dyes, simplifying high throughput screening. Thus, this technology is suitable for sensitive measurements of various cellular events such as live cell imaging, cell migration, proliferation, differentiation, and death [31,33,34,35,36,37,38,39]. Identifying and measuring specific morphological features by DHM during early stages of exposure to toxic agents such as ricin and abrin, could allow early cell death detection, thereby determining toxin activity. As a label-free technique, DHM does not require pipetting steps which can affect the cell and even induce strong bias as some detached dead cell can be washed out. We therefore set out to develop an activity assay for ricin and abrin based on DHM evaluation of cells following intoxication.

In this study, we present the feasibility of a cell-based assay for ricin and abrin intoxication using DHM. In contrast to current methodologies, DHM is noninvasive, rapid and able to monitor changes in cell morphology dynamically, allowing early detection of low dose intoxication. The method described herein may be applied for early and sensitive detection of these as well as other toxins and substances.

## 2. Results

### 2.1. Morphological Changes in Cell Lines Observed by DHM Following Ricin Exposure

In order to characterize morphological changes mediated by ricin exposure, HeLa cells were seeded in six-well plates at different confluence levels (10–30%) and incubated for 4–6 h for cell adhesion. Following incubation, cells were exposed to ricin at 100 ng/mL and digital holograms of four different areas in each well were recorded at 10 min time intervals for 20 h.

**Figure 1:**
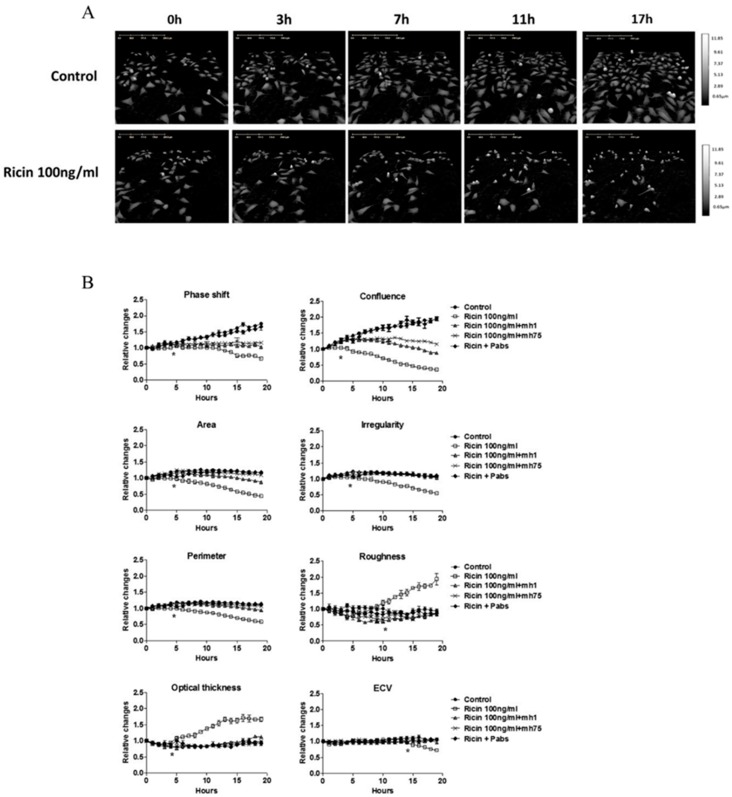
Morphology features of ricin intoxication detected by digital holographic microscopy (DHM). Effect of ricin treatment on HeLa cells was tested using DHM. Cells were subjected to ricin (100 ng/mL), and digital holograms of four different areas in each well were recorded for 20 h at 10 min intervals. Untreated cells were used as a control. (**A**) Representative three-dimensional Images of treated and untreated cells as captured at 0, 3, 7, 11, and 17 h. Marker for optical thickness depicted on the right side. (**B**) Quantification of the relative changes in various morphological parameters was performed in parallel in the presence of neutralizing antibodies MH1 (monoclonal anti-subunit A), MH75 (monoclonal anti-subunit B), and polyclonal antibodies. * indicate *p* < 0.05 of intoxicated vs. untreated cells according to 2-tailed Student’s *t*-test.

The resulting digital holographic images were used to measure and define changes in various morphological and optical features of treated versus untreated cells. Images of treated cells depicted in Figure 1A show a representative 3D holographic images of HeLa cells treated with 100 ng/mL ricin. A visual inspection of the images shows a decrease in cell numbers and confluence within 7 to 11 h and increased cell thickness and roundness within 11 h post-ricin exposure compared to untreated cells. Increased cell thickness and roundness following ricin exposure, indicates that the cells undergo apoptosis or detachment from the plate. These differences between treated and untreated cells were quantified by the DHM. Results (Figure 1B) show that significant changes occur within 3 to 6 h post-ricin exposure in confluence, irregularity, and optical cell thickness. Moreover, DHM enables the monitoring of additional parameters including phase-shift, cell area, perimeter, roughness, and effective-calculated volume (ECV) which could not be observed visually. In addition, the relative changes in cell counts measured by DHM support the visual inspection indicating a marked decrease in cell divisions (Figure S1). In general, significant changes were observed within 4–7 h post-ricin intoxication.

To ascertain whether these changes are attributed specifically to ricin intoxication, neutralizing polyclonal antibodies against ricin were added to cells concomitantly with toxin administration. As shown in Figure 1B, the remarkable morphological changes described above were completely inhibited. This verifies that these changes are specifically mediated by ricin. In order to find out whether these morphological changes are mediated by the presence of ricin in the cell or its catalytic activity, we used monoclonal antibodies for blocking either the entrance of the ricin to the cell (MH75, anti-subunit B) or the catalytic activity of ricin (MH1 anti-subunit A) [6,40]. As shown in Figure 1B, no changes in morphology parameters were observed by blocking ricin either by MH1 or MH75 antibodies. A complete inhibition was observed in inducing changes in parameters such as area, irregularity, perimeter, roughness, optical thickness and the ECV. Confluence and phase-shift were slightly decreased apparently due to traces of unblocked ricin, suggesting these parameters are highly sensitive and more pronounced in cases of low concentrations of toxin exposure. Overall, these findings show that morphological and optical changes in intoxicated cells are related specifically to the catalytic activity of ricin.

**Figure 2:**
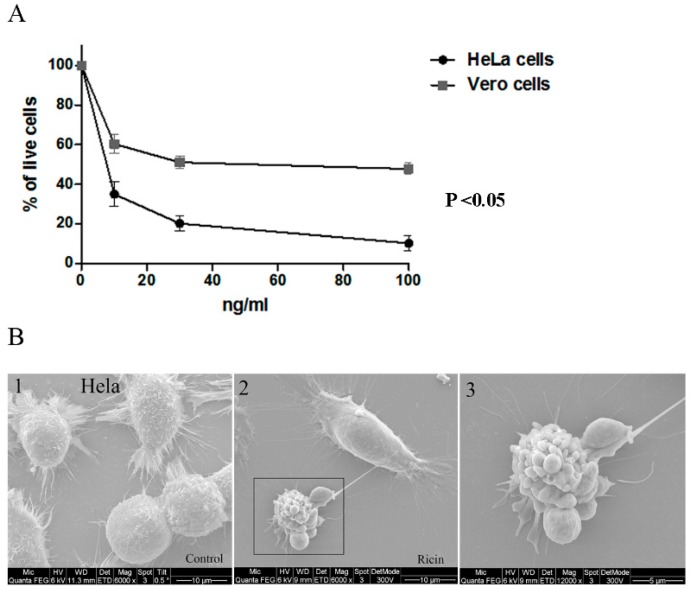
The effect of ricin intoxication on cell viability. HeLa and Vero cells were incubated in the presence and absence of the toxin at concentrations of 10–100 ng/mL. (**A**) AlamarBlue viability assays were performed 17 h post-ricin exposure. The percentage of viable cells (mean ± SD) in treated cells was calculated relatively to untreated cells in each measurement. *p* < 0.05 of HeLa vs. Vero-treated cells was calculated according to 2-tailed Student’s *t*-test. (**B**) Scanning electron microscopy imaging of ricin intoxication on HeLa cells morphology was performed 5 h post 100 ng/mL ricin exposure. Panel 1 represents untreated cells, panel 2 represents ricin treated cells, and panel 3 represents higher magnification of the inset in panel 2.

In order to define the suitable cell line in terms of assay sensitivity, as well as other technical aspects, the Vero cell line was chosen in lieu of the fact that this cell line (similar to HeLa cells) is widely used in the study of ricin intoxication and neutralization [20,41]. Table 1 summarizes the time ranges for the detection of significant changes (*p* < 0.05) in morphological features compared to untreated cells for the two cell lines. These results summarize several independent experiments (n = 3), with some variation in terms of cell initial confluence and adhesion times before toxin was administered. The same trend of morphological changes was observed for both cell lines compared to HeLa cells. Vero cells were less sensitive to 100 ng/mL ricin compared to HeLa cells, which manifested in a significant delay in morphology change detection. The only exception was ECV, which was significantly reduced in Vero cells within 4 to 7 h compared to 14–15 h in HeLa cells. In order to verify whether the observed morphological changes during intoxication of HeLa and Vero cells are related to cell death, an established viability assay using AlamarBlue, was performed in a dose–response assay. As shown in Figure 2A, a 90% decrease in cell viability was observed within 17 h of intoxication of HeLa cells, while a reduction of 50% was observed at that time point for intoxicated Vero cells.

The differences in structural features during toxic exposure were visualized using scanning electron microscopy (Figure 2B). Five hours post-ricin exposure more apoptotic cells were observed, identified by increased cell roundness and the appearance of blebbing in cell membranes, which might correlate with the increased optical thickness and roughness observed in DHM.

### 2.2. Similarities in Morphological Features during Abrin Toxicity

**Figure 3:**
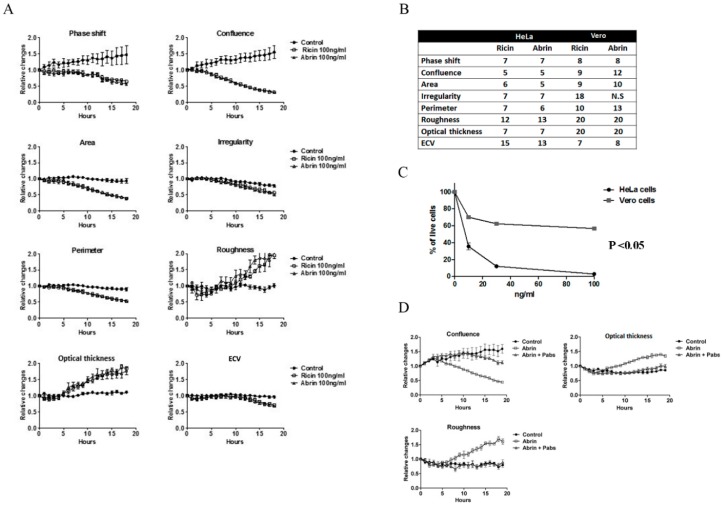
Similarities in morphology features during ribosome inactivating proteins (RIPs) intoxication. Comparison of ricin and abrin intoxication on various morphology features in HeLa and Vero cell lines. HeLa (A–C) and Vero (B–C) were treated with ricin and abrin (100 ng/mL) and digital holograms of four different areas in each well were recorded every 10 min for 19 h. Untreated cells were used as a control. (**A**) Quantification of the relative changes in morphological parameters (mean ± SE) detected using DHM. (**B**) The table summarizes the time range for the detection of significant changes in the morphological features followed of intoxicated HeLa and Vero cells compared to untreated cells. *p* < 0.05 was calculated according to 2-tailed Student’s *t*-test. (**C**) Effect of abrin on viability of HeLa and Vero cells was performed 17 h post-toxin exposure (10–100 ng/mL) using AlamarBlue assay. The percentage of viable cells (mean ± SD) in treated cells were calculated relatively to untreated cells in each measurement. *p* < 0.05 of HeLa- vs. Vero-treated cells was calculated according to 2-tailed Student’s *t*-test. (**D**) Confluence, roughness, and optical thickness are representative parameters show neutralizing effect of anti-abrin polyclonal antibodies (pAbs) on abrin intoxicated HeLa cells detected by DHM.

Since ricin and abrin share high structure homology as well as the same biological activity, we tested whether their toxic effect in vitro will be similar. To determine if this is the case, a comparison of the toxic effect of ricin and abrin (100 ng/mL) was performed. As expected, the same trend in morphological changes was observed, with no significant differences in time ranges (Figure 3A,B). As was shown for ricin (Table 1), HeLa cells exhibited earlier significant morphological changes following intoxication and a significant reduction in cell viability when compared to Vero cells (Figure 3C). In addition, these changes were inhibited by adding neutralizing anti-abrin polyclonal antibodies (Figure 3D). In agreement with Ricin intoxication (Table 1), the ECV of Vero cells was reduced significantly earlier during abrin exposure, suggesting ECV as one of the most sensitive parameters in Vero cells to be affected during cell toxicity detected by DHM. Despite significant changes observed in ECV of intoxicated Vero cell, we decided to continue our assay development with HeLa cells since they exhibits significantly more and earlier distinct phenotypical changes.

### 2.3. Early Morphological Changes Detected by DHM Are Attributed to Live Cells

**Figure 4:**
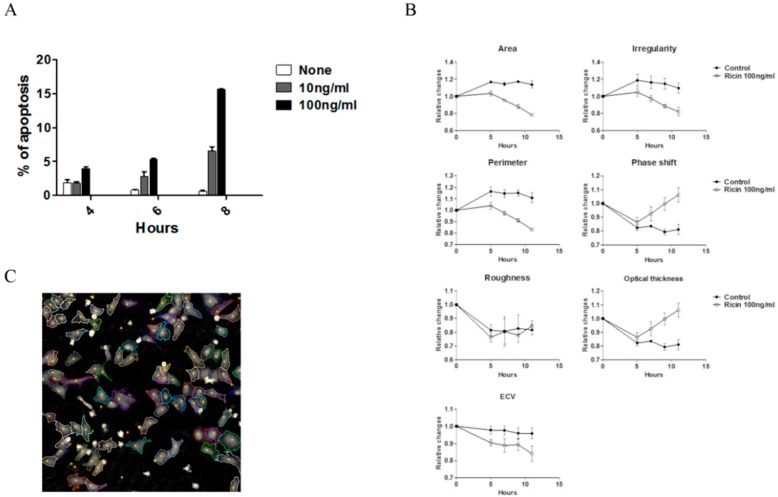
Intoxication evaluation of live cells by DHM. HeLa cells were exposed to ricin 10–100 ng/mL. (**A**) Percentage of apoptotic cells (mean ± SE) during 8 h of ricin intoxication was determined using annexin V and PI apoptosis assays. (**B**) Live cell segmentation was performed using DHM cell tracking application according to roundness and thickness parameters in each frame. In each frame, 50–60 cells were tracked and analyzed. Treated and untreated tracked live cells were analyzed for morphological changes during 11 h post-ricin exposure (100 ng/mL). Shown are representative results out of three independent experiments (mean of 4 different areas in each well ± SE). (**C**) A representative DHM 2D image for presumed live cell segmentation analysis (cell margins are highlighted by colored lines).

Thus far, we demonstrated that DHM allows for morphological analysis that can identify significant changes induced by ricin/abrin intoxication by analyzing the entire field of view. The cell populations analyzed are comprised of cells undergoing all stages of ricin intoxication, including early and late apoptotic cells. As a consequence, each parameter is an average of a heterogenic cell population. Using the annexin V and PI apoptotic assay we could evaluate the early and late apoptotic cell populations reaches ~15% within 8 h post 100 ng/mL ricin exposure (Figure 4A and Figure S2A). The apoptotic cells, exhibiting annexin V+/PI+ double staining, reflect late apoptotic stages. These cells show also significant changes in size and granularity detected by the forward and side scatter (FSC/SSC) parameters (Figure S2B). In an attempt to focus on the morphological changes of the live cell fraction (undergoing early intoxication stages), we analyzed individual cells in each frame by using the ’tracking cells’ application. This allows the exclusion of presumed dead cells defined by parameters such as roundness and thickness (Figure 4B). We presume that these cells are the late apoptotic cells defined by flow cytometry in Figure 4A and Figure S2. Next, we analyzed the remaining cell population (live cells and early apoptotic cells) for morphological changes. Analysis of these cells during the first 11 h post-ricin exposure shows the same trend in morphological parameters as previously described, starting 5 h and increasing with time up to 11 h after intoxication (Figure 4C). These results show that morphological changes after intoxication are manifested also by cells in early stages of intoxication and are not fully related or biased by the late stage of intoxicated cells. Interestingly, the roughness feature was unaffected in treated tracked cells, suggesting roughness is in fact a specific indicator of late apoptotic outcome. Detection of morphological changes in live cells upon early stages of intoxication emphasizes the high sensitivity of this method for assessing toxin activity.

### 2.4. Increased Susceptibility to RIP Intoxication during Cell Adhesion

**Figure 5:**
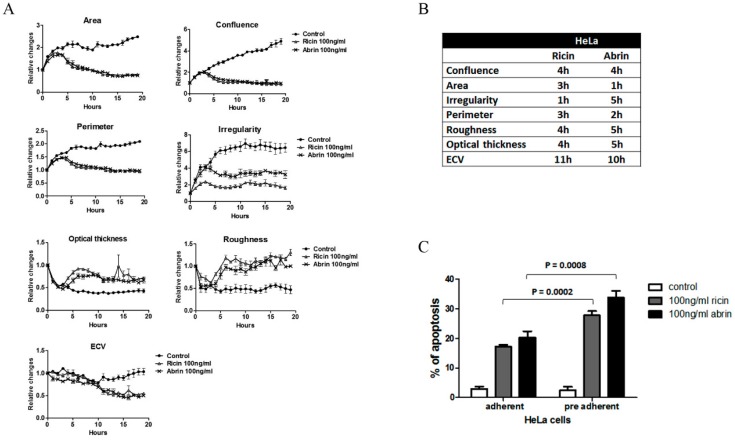
Increased susceptibility of preadherent versus adherent HeLa cells to RIP intoxication. HeLa cells were expose to ricin or abrin in two modes of intoxication: precell adhesion and post-cell adhesion. The effect of the state of the cells on ricin and abrin intoxication was evaluated by two approaches: DHM and Annexin V/PI assays. (**A**) DHM analysis of the relative changes in morphological parameters in preadherent intoxicated HeLa cells (mean ± SE). (**B**) Table summarizes the time range for the detection of significant changes in the morphological features as presented in A. *p* < 0.05 was calculated according to 2-tailed Student’s *t*-test. (**C**) Annexin V and PI apoptosis assays at 8 h postintoxication (mean ± SE). *p* value of preadherent vs. adherents cells for ricin or abrin intoxication were calculated according to 2-tailed Student’s *t*-test.

Having performed all of our experiments on adherent HeLa cells, we wanted to test the possibility of intoxicating cells and monitoring morphological effects on preadherent HeLa cells. Such a change in protocol could allow shortening the assays by 4 to 6 h, provided that sensitivity is not compromised. In order to do so, HeLa cells were seeded (5 × 10^4^/mL, 3 mL) in six well plates, following toxin administration without preincubation for cell adhesion. Digital holograms of four different areas in each well were recorded at 10 min time intervals. In a visual inspection of the preadherent cells, sedimentation and spreading occur within 3 h, both in treated and untreated cells (Figure S3). Seven hours postincubation, most of the intoxicated cells were defined as round cells with increased optical thickness. Quantification by DHM analysis showed that skipping the 4–6 h of cell adhesion increased assay sensitivity, with morphological changes consistent with those described above, but detected by the DHM earlier than the changes in fully adhered cells. This was true for both ricin and abrin treatments (100 ng/mL) (Figure 5A,B and Table 2). Interestingly, cell roughness was the most affected parameter by the new protocol, resulting in significant elevation as early as 4 h after intoxication. This reflects the high sensitivity of preadherent HeLa cells to ricin as was confirmed by apoptosis assays of adherent vs. preadherent cells (Figure 5C). In addition, beginning our morphological monitoring at this early stage means that the differences between untreated cells and intoxicated cells were more pronounced over time, increasing the sensitivity of the assay. Similarly to previous results (Figure 3), the morphological changes induced by exposure to different concentrations of ricin and abrin (10–100 ng/mL) were similar (Figure 5A and Figure S4). This observation was also confirmed by the apoptotic assay (Figure 5C).

**Figure 6:**
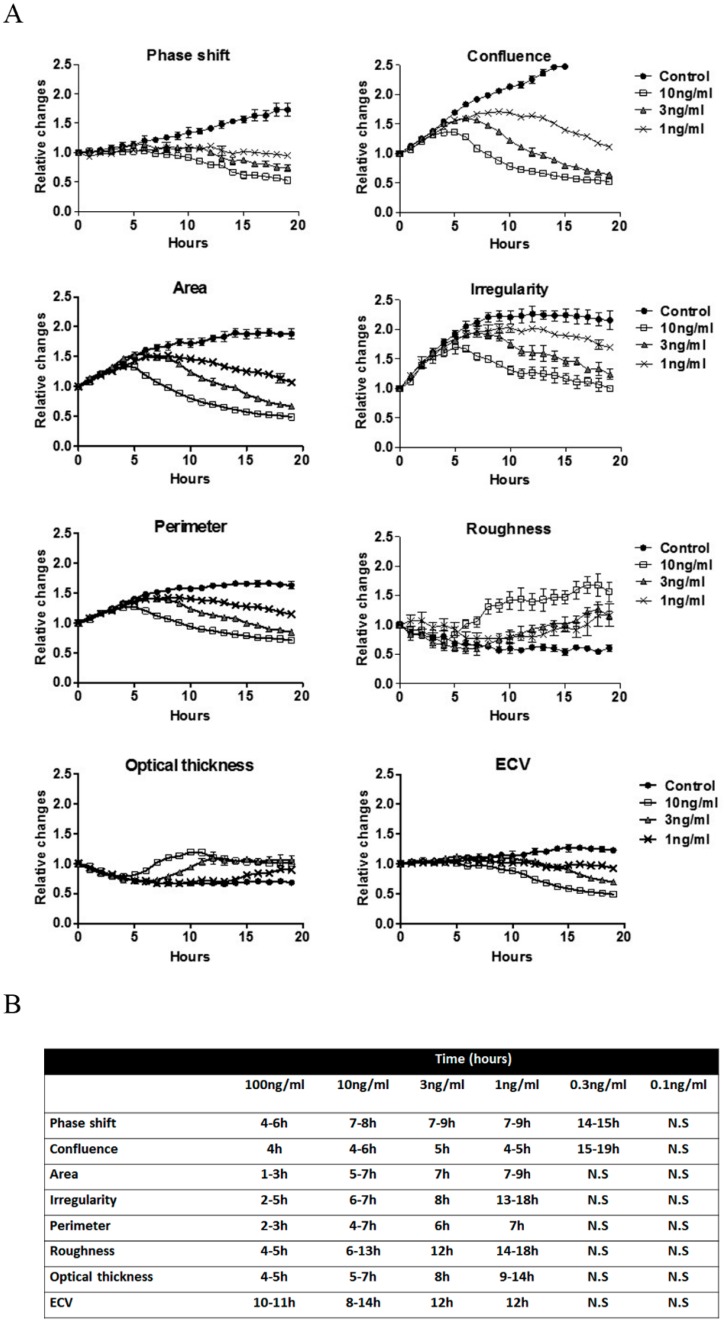
A dose–response curve of ricin intoxication during cell adhesion. Preadherent HeLa cells were treated with 0.1–100 ng/mL ricin and (**A**) digital holograms of 4 different areas in each well were recorded every 10 min. A representative quantification of the relative changes in morphological parameters detected by DHM presented as mean ± SE. (**B**) Table summarizes the time ranges for the detection of significant changes (*p* < 0.05 according to 2-tailed Student’s *t*-test) in the morphological features followed of intoxicated HeLa cells compared to untreated cells. Values are taken from several independent experiments. N.S.-non significant.

A dose–response assay for preadherent HeLa cells was performed at 0.1–100 ng/mL (Figure 6). Following 10 ng/mL ricin exposure, changes in confluence, area, perimeter, and optical thickness were observed 4–5 h after intoxication, while phase-shift, irregularity, and roughness changed after 6–7 h. At lower concentrations of toxins, as expected, a slight delay in the development of morphological changes was observed. Interestingly, confluence, and phase-shift at 1 ng/mL ricin were remarkably decreased after 4–5 and 7–9 h, respectively. These results support our previous observation, depicted in Figure 1B, that these parameters are highly sensitive and more pronounced in cases of low concentrations of toxin exposure.

## 3. Discussion

Digital holographic microscopy (DHM) is a time-lapse, label-free, and noninvasive imaging technique that can provide both qualitative and quantitative phase information on morphological features of cells. Detection by DHM of morphological features characteristic of early stages of cell death [37] can provide a rapid and sensitive method for assessing cell cytotoxicity upon interaction with drugs, toxins and interfering compounds.

In this study, we employed DHM to evaluate the morphological changes of cell lines during ricin and abrin intoxication. We demonstrated that the effect of ricin and abrin intoxication on HeLa and Vero cell lines is characterized by a decrease in cell confluence which was also observed by monitoring less cell divisions and a reduction of total cell counts compared to untreated cells. In addition, a decrease in morphological parameters such as cell area, perimeter, irregularity, and ECV, and an increase in optical thickness and roughness were also observed. These effects are directly attributed to ricin or abrin intoxication as they were inhibited by the addition of neutralizing antibodies against ricin or abrin respectively.

The morphological changes analyzed by the DHM are related to the total cell population. To exclude the contribution of dead cells from the analysis we used the cell tracking application of the DHM. This allowed us to examine the effect of ricin on live cells exclusively. Detection of morphological changes in presumed live cells upon early stages of intoxication reflects a rapid and sensitive method for assessing toxin activity. It was found that HeLa cells exhibited earlier significant morphological changes following intoxication and a significant reduction in cell viability, when compared to Vero cells.

Shortening the preparation time of the assay by exposing preadherent HeLa cells to toxins increased assay sensitivity, with an earlier onset of detectable morphological changes compared to fully adhered cells. A further reduction of toxin concentrations was performed in a dose–response assay (0.1–100 ng/mL), showing clear detection of morphological changes within 4 to 6 h for 10 ng/mL toxins. Significant changes in confluence and phase-shift parameters were also observed in 0.3 ng/mL intoxication within 15 h, defining the limit of detection of the assay. The effect of cell adhesion on drug- induced apoptosis was well described in several studies [42,43,44]. Damiano et al. (1999) demonstrated the critical role of integrins and extracellular matrix interaction in cell survival and showed that cells in direct contact with the integrin fibronectin are less sensitive to acute doxorubicin treatment.

The addition of anti-ricin polyclonal antibodies induced a complete inhibition of ricin-induced morphological changes, while the use of the monoclonal antibodies (either directed against subunit A or subunit B) induced only a partial inhibition of cell confluence and phase-shift and complete inhibition of all other parameters. These variations may originate from the fact the polyclonal antibodies are composed of antibodies that target different epitopes simultaneously, increasing the neutralization effect.

The DHM in our assay was able to detect single cells for analysis only at confluence levels of under 70%. Above this level, an inconsistency in consecutive frame analyze was observed, hampering the analysis after 20 h of incubation (due to cell replication). In order to detect changes in HeLa and Vero cells with a low dose of toxins—less than 0.3 ng/mL—prolonged intoxication times are crucial. To that end, cells should be initially cultured at less than 10% confluence. Another option, not tested in this study, is to limit cell replication (by serum starvation, etc.).

The characterization of toxin-mediated morphology changes of single cells, detected by DHM, was also examined with shiga toxin [45], resulting in cell rounding and increased cell thickness. Interestingly, morphological changes observed upon etoposide intoxication occur prior to the reduction in cell metabolic activity or viability as measured by MTS assay [46]. In addition, monitoring cell cycle arrest [35], and cytotoxicity assessment [31,37] by DHM were found comparable to established assays such as flow cytometry, fluorescence-based methods, and trypan blue assays.

Phototoxicity has the ability to increase the intoxication effect of ricin, as was demonstrated by Song et al. [47] using fluorescent microscopy for cell imaging, thus raising a question regarding similar effects in the DHM setup. The phototoxicity level in DHM was addressed by several groups who have demonstrated that the light intensity is six order of magnitude lower than intensities typically associated with confocal fluorescent microscopy and well below the phototoxicity levels [37,38,48]. The advantage of DHM is that it is a label-free technique that does not requires the additions of dyes that by interacting with the light might exacerbate the toxin effects. Additionally, we have demonstrated that by adding toxin-neutralizing antibodies we have completely inhibited all morphological and optical effects, thus implying that phototoxicity-induced nonspecific effects following ricin toxicity are minimal if any.

Cell based assays for ricin toxicity measure cell survival as the intoxication end point, usually within 48 to 72 h postexposure [16,17]. As ricin-mediated protein synthesis arrest is an early cellular event, it can be detected in vitro by measuring marker protein levels 3–24 h postintoxication [6,18,19,20]. In this study, we demonstrate that significant changes in cellular optical and morphology parameters occur as early as 4–6 and 2–3 h using 10 and 100 ng/mL of ricin, respectively. These results are well correlated to previous reports that measured inhibition of intracellular protein synthesis using the same toxin concentrations, and therefore strengthen the use of DHM to study and follow ricin activity. Moreover, the use of specific antitoxin neutralizing antibodies to inhibit the toxic effects on the cells is crucial in every cell-based as well as noncell-based assay. This an internal control for the specificity of the assays. When compared to other cell-based methods, DHM does not requires any additional steps to evaluate the toxin effect, thus making this method simple yet sensitive.

Overall, our results emphasize the benefits of using DHM for the detection of ricin and abrin activity in cell lines as a rapid and sensitive assay. Application of cell morphology detection in high throughput screening is used by researchers in academia and pharma in order to address cell death determination in drug discovery [49,50], adverse cellular effects [51], venom exploration [52], and is also in common use in the clinical setting for the detection of *C. difficile* toxins [53].

The ability of DHM technology to detect early changes in cellular features upon toxin-mediated cell death processes makes it suitable for toxin activity assays, and is comparable, if not superior, to established methods.

## 4. Materials and Methods

### 4.1. Toxins and Antibodies

Purified ricin and abrin were prepared as described previously [54,55]. Anti-ricin subunit A (MH1) and anti-subunit B (MH75) in-house monoclonal antibodies utilized in this study were produced from nonhuman primates [40]. In-house anti-ricin and anti-abrin polyclonal antibodies were obtained from immunized rabbit serum [54,55].

### 4.2. Cell Lines

HeLa and Vero cell lines were obtained from the American Type Culture Collection (ATCC). Cells were cultured in Dulbecco’s modified Eagle’s medium (DMEM) supplemented with 10% (*v*/*v*) fetal bovine serum and 1% L-glutamine (200 nM) and incubated at 37 °C in 5% CO_2_ humidified incubator.

### 4.3. Digital Holographic Microscopy

Digital holographic microscopy was performed using a Holomonitor M4 microscope (Phase Holographic Imaging AB, Lund, Sweden). The Holomonitor M4 is a label-free cell analyzer for adherent cells equipped with X20 magnification objective (NA-0.4), a 0.8 mW HeNe laser (633 nm, 100 µW/cm^2^, exposure time 5 ms), and a motorized stage. Digital holographic microscopy, as a quantitative phase-contrast imaging method, is a form of optical interferometry, which detects the phase delay related to the light passing through the tested object. Images were converted from wavelength interaction to cells’ representation by a computer algorithm (Hstudio 2.6, Phase Holographic Imaging AB, Lund, Sweden). This computation enables to obtain information on various morphological and optical parameters. Two different threshold settings prior to segmentation of cells were used for analyzing the experiments: Otsu thresholding (OT) [56] and minimum error thresholding (MET) [57]. While the former results in a more accurate identification of cells, by excluding focus debris, MET gave a more accurate cell outline. In our study, the same trend was observed using the two threshold settings and the DHM results presented in this study were analyzed according to MET threshold. In general, cell lines (1–4 × 10^5^) were seeded in 6-well plates at different degrees of confluence (10–30%) with or without incubation for 4 to 6 h for cell adhesion. Following incubation, cells were exposed to ricin or abrin at 1–100 ng/mL in a 3 mL volume. The neutralizing effect of anti-ricin and anti-abrin antibodies was assessed by incubating toxins with 20 μg MH1 or MH75 monoclonal antibodies or 1:100 dilution of polyclonal antibodies. Cells were monitored continuously, in time-lapse mode (every 10 min), in multiple locations in each well using a high precision motorized stage. Quantified parameters obtained from DHM in each frame were analyzed as changes relative to time zero from each area and presented as mean ± SE for all the monitored areas in each well. In this work we focused on significant changes observed during intoxication in several parameters: phase-shift (phase delay related to the light passing through the tested object), confluence, area, irregularity (determines the deviation of the cell region from the circular shape), perimeter, and roughness (indication on the smoothness of the surface of the cell). As the refractive index of the cell and the surrounding medium cannot be measured, the presented cell thickness and volume values are expressed as the optical thickness and effective-calculated volume (ECV).

### 4.4. Viability Assay

The cytotoxic effects of purified ricin and abrin on HeLa and Vero cells were determined by AlamarBlue viability assay (Promega, Madison, WI, USA). Briefly, cells were cultured in 96-well plates overnight at a density of approximately 10^4^/well and then treated with 10–100 ng/mL of ricin or abrin for 3–17 h. AlamarBlue reagent was added to each well up to 10% of tissue culture medium and incubated for an additional 3 at 37 °C. Cell viability was determined by measuring absorbances at 562 nm and 600 nm.

### 4.5. Flow Cytometry-Apoptosis Assay

Apoptosis induction with ricin and abrin in HeLa cells was assessed by APC Annexin V/PI apoptosis kit (catalog #460932, BioLegend, San Diego, CA, USA) and analyzed using a BD Fortesa Flow Cytometer from Becton Dickinson (San Jose, CA, USA). In general, 5 × 10^4^/well HeLa cells were cultured overnight in 24-wells plates for cell adhesion. Following cell adhesion, toxins (10–100 ng/mL) were added to cells and apoptosis measurements were performed after 4–8 h. In order to assess the effect of cell adhesion on toxin susceptibility, HeLa cells were treated with trypsin for cell detachment and washed in a fresh growth medium prior to toxin administration. Apoptosis induction was compared between the preadherent and fully adherent cells. Untreated preadherent/adhered cells were used as a control.

### 4.6. Scanning Electron Microscopy

HeLa cells were grown in 75 cm^2^ flasks at 37 °C and 5% CO_2_. The cells were detached using trypsin and then seeded in 24 well plates at ~50,000 cells/well on top of polylysine-coated cover slips. The cells were allowed to attach and treated with ricin for 5 h at 37 °C. After incubation, the cells were washed with phosphate-buffered saline (PBS) once and a fixative buffer containing 2.5% glutaraldehyde and 2% paraformaldehyde in 0.1 M cacodylate buffer (Cat. 15960-01, EMS, Hatfield, PA, USA) was added. Cells were washed in PBS and 1% osmium tetraoxide was added for 1 h at room temperature (RT) Cells were washed in DDW and dehydration was carried out in a gradient of ethanol concentrations. After dehydration, the ethanol was substituted with CO_2_ in a Critical Point Drying (CPD, Ashford Kent, UK) machine and, finally, the cover slips containing cells were coated with 2 nm iridium. The cells were imaged using QuantaFEG SEM (Thermo Fisher, OR, USA) operated at 6 kV in high vacuum mode using an ET detector.

### 4.7. Statistical Analysis

All experiments were repeated three times and data was analyzed using GraphPad Prism5 software (San Diego, CA, USA). Results are expressed as mean ± standard error. Statistical significance was determined by 2-tailed unpaired Student’s *t*-test. *p* value ≤ 0.05 was considered to be significant.

## Figures and Tables

**Table 1:** Statistical significance changes in morphology features detected by DHM.

	Time (h)	
	HeLa	Vero
Phase-shift	5–7	7–8
Confluence	3–5	9
Area	4–6	6–9
Irregularity	4–7	16–18
Perimeter	4–7	6–10
Roughness	11–12	18–20
Optical thickness	4–7	17–20
ECV	14–15	4–7

*p* < 0.05.

**Table 2:** Statistical significance changes in morphology features detected by DHM in adherent compared to preadherent cells.

	Time (h)	
	Adherent HeLa Cells	Preadherent HeLa Cells
Phase-shift	5–7	4–6
Confluence	3–5	4
Area	4–6	1–3
Irregularity	4–7	2–5
Perimeter	4–7	2–3
Roughness	11–12	4–5
Optical thickness	4–7	4–5
ECV	14–15	10–11

*p* < 0.05.

## Supplementary Materials

The following are available online at https://www.mdpi.com/2072-6651/11/3/174/s1. Figure S1: Reduction in cell count by ricin intoxication measured by DHM. Figure S2: The induction of cell death by ricin determined by Annexin V/PI apoptosis assay. Figure S3: Morphology features of ricin intoxication of preadherent HeLa cells detected by DHM. Figure S4: Similarities in morphology features during RIPs intoxication.

## References

[1] Stirpe F. (2004). Ribosome-inactivating proteins. Toxicon.

[2] Sperti S., Montanaro L., Mattioli A., Testoni G. (1975). Relationship between elongation factor I- and elongation factor II- dependent guanosine triphosphatase activities of ribosomes. Inhibition of both activities by ricin. Biochem. J..

[3] Olivieri F., Prasad V., Valbonesi P., Srivastava S., Ghosal-Chowdhury P., Barbieri L., Bolognesi A., Stirpe F. (1996). A systemic antiviral resistance-inducing protein isolated from Clerodendrum inerme Gaertn. is a polynucleotide:adenosine glycosidase (ribosome-inactivating protein). FEBS Lett..

[4] Stirpe F., Barbieri L., Battelli M.G., Soria M., Lappi D.A. (1992). Ribosome-inactivating proteins from plants: Present status and future prospects. Biotechnology (N Y).

[5] Endo Y., Tsurugi K. (1987). RNA N-glycosidase activity of ricin A-chain. Mechanism of action of the toxic lectin ricin on eukaryotic ribosomes. J. Biol. Chem..

[6] Gal Y., Alcalay R., Sabo T., Noy-Porat T., Epstein E., Kronman C., Mazor O. (2015). Rapid assessment of antibody-induced ricin neutralization by employing a novel functional cell-based assay. J. Immunol. Methods.

[7] Iordanov M.S., Pribnow D., Magun J.L., Dinh T.H., Pearson J.A., Chen S.L., Magun B.E. (1997). Ribotoxic stress response: Activation of the stress-activated protein kinase JNK1 by inhibitors of the peptidyl transferase reaction and by sequence-specific RNA damage to the alpha-sarcin/ricin loop in the 28S rRNA. Mol. Cell Biol..

[8] Saenz J.B., Doggett T.A., Haslam D.B. (2007). Identification and characterization of small molecules that inhibit intracellular toxin transport. Infect. Immun..

[9] Brigotti M., Barbieri L., Valbonesi P., Stirpe F., Montanaro L., Sperti S. (1998). A rapid and sensitive method to measure the enzymatic activity of ribosome-inactivating proteins. Nucleic Acids Res..

[10] Chen X.Y., Link T.M., Schramm V.L. (1998). Ricin A-chain: Kinetics, mechanism, and RNA stem-loop inhibitors. Biochemistry.

[11] Falach R., Sapoznikov A., Gal Y., Israeli O., Leitner M., Seliger N., Ehrlich S., Kronman C., Sabo T. (2016). Quantitative profiling of the in vivo enzymatic activity of ricin reveals disparate depurination of different pulmonary cell types. Toxicol. Lett..

[12] Hines H.B., Brueggemann E.E., Hale M.L. (2004). High-performance liquid chromatography-mass selective detection assay for adenine released from a synthetic RNA substrate by ricin A chain. Anal. Biochem..

[13] Li M., Pestka J.J. (2008). Comparative induction of 28S ribosomal RNA cleavage by ricin and the trichothecenes deoxynivalenol and T-2 toxin in the macrophage. Toxicol. Sci..

[14] Stirpe F., Bailey S., Miller S.P., Bodley J.W. (1988). Modification of ribosomal RNA by ribosome-inactivating proteins from plants. Nucleic Acids Res..

[15] Taylor B.E., Irvin J.D. (1990). Depurination of plant ribosomes by pokeweed antiviral protein. FEBS Lett..

[16] Prigent J., Panigai L., Lamourette P., Sauvaire D., Devilliers K., Plaisance M., Volland H., Creminon C., Simon S. (2011). Neutralising antibodies against ricin toxin. PLoS ONE.

[17] Vance D.J., Tremblay J.M., Mantis N.J., Shoemaker C.B. (2013). Stepwise engineering of heterodimeric single domain camelid VHH antibodies that passively protect mice from ricin toxin. J. Biol. Chem..

[18] Cohen O., Mechaly A., Sabo T., Alcalay R., Aloni-Grinstein R., Seliger N., Kronman C., Mazor O. (2014). Characterization and epitope mapping of the polyclonal antibody repertoire elicited by ricin holotoxin-based vaccination. Clin. Vaccine Immunol..

[19] Halter M., Almeida J.L., Tona A., Cole K.D., Plant A.L., Elliott J.T. (2009). A mechanistically relevant cytotoxicity assay based on the detection of cellular GFP. Assay Drug. Dev. Technol..

[20] Wahome P.G., Ahlawat S., Mantis N.J. (2012). Identification of small molecules that suppress ricin-induced stress-activated signaling pathways. PLoS ONE.

[21] Duprez L., Wirawan E., Vanden Berghe T., Vandenabeele P. (2009). Major cell death pathways at a glance. Microbes Infect.

[22] Krysko D.V., Vanden Berghe T., D’Herde K., Vandenabeele P. (2008). Apoptosis and necrosis: Detection, discrimination and phagocytosis. Methods.

[23] Rao P.V., Jayaraj R., Bhaskar A.S., Kumar O., Bhattacharya R., Saxena P., Dash P.K., Vijayaraghavan R. (2005). Mechanism of ricin-induced apoptosis in human cervical cancer cells. Biochem. Pharmacol..

[24] Saxena N., Yadav P., Kumar O. (2013). The Fas/Fas ligand apoptotic pathway is involved in abrin-induced apoptosis. Toxicol. Sci..

[25] Lecoeur H. (2002). Nuclear apoptosis detection by flow cytometry: Influence of endogenous endonucleases. Exp. Cell Res..

[26] Zamai L., Falcieri E., Marhefka G., Vitale M. (1996). Supravital exposure to propidium iodide identifies apoptotic cells in the absence of nucleosomal DNA fragmentation. Cytometry.

[27] Korzeniewski C., Callewaert D.M. (1983). An enzyme-release assay for natural cytotoxicity. J. Immunol. Methods.

[28] Neumann B., Held M., Liebel U., Erfle H., Rogers P., Pepperkok R., Ellenberg J. (2006). High-throughput RNAi screening by time-lapse imaging of live human cells. Nat. Methods.

[29] Strbkova L., Zicha D., Vesely P., Chmelik R. (2017). Automated classification of cell morphology by coherence-controlled holographic microscopy. J. Biomed. Opt..

[30] Khmaladze A., Matz R.L., Epstein T., Jasensky J., Banaszak Holl M.M., Chen Z. (2012). Cell volume changes during apoptosis monitored in real time using digital holographic microscopy. J. Struct. Biol..

[31] Kühn J., Shaffer E., Mena J., Breton B., Parent J., Rappaz B., Chambon M., Emery Y., Magistretti P., Depeursinge C. (2013). Label-free cytotoxicity screening assay by digital holographic microscopy. Assay Drug Dev. Technol..

[32] Molder A., Sebesta M., Gustafsson M., Gisselson L., Wingren A.G., Alm K. (2008). Non-invasive, label-free cell counting and quantitative analysis of adherent cells using digital holography. J. Microsc..

[33] Bettenworth D., Lenz P., Krausewitz P., Bruckner M., Ketelhut S., Domagk D., Kemper B. (2014). Quantitative stain-free and continuous multimodal monitoring of wound healing in vitro with digital holographic microscopy. PLoS ONE.

[34] El-Schich Z., Molder A., Tassidis H., Harkonen P., Falck Miniotis M., Gjorloff Wingren A. (2015). Induction of morphological changes in death-induced cancer cells monitored by holographic microscopy. J. Struct. Biol..

[35] Falck Miniotis M., Mukwaya A., Gjorloff Wingren A. (2014). Digital holographic microscopy for non-invasive monitoring of cell cycle arrest in L929 cells. PLoS ONE.

[36] Marquet P., Rappaz B., Magistretti P.J., Cuche E., Emery Y., Colomb T., Depeursinge C. (2005). Digital holographic microscopy: A noninvasive contrast imaging technique allowing quantitative visualization of living cells with subwavelength axial accuracy. Opt. Lett..

[37] Pavillon N., Kuhn J., Moratal C., Jourdain P., Depeursinge C., Magistretti P.J., Marquet P. (2012). Early cell death detection with digital holographic microscopy. PLoS ONE.

[38] Rappaz B., Breton B., Shaffer E., Turcatti G. (2014). Digital holographic microscopy: A quantitative label-free microscopy technique for phenotypic screening. Comb. Chem. High Throughput Screen.

[39] Fang Y. (2011). Label-free biosensors for cell biology. Int. J. Electrochem..

[40] Noy-Porat T., Rosenfeld R., Ariel N., Epstein E., Alcalay R., Zvi A., Kronman C., Ordentlich A., Mazor O. (2016). Isolation of Anti-Ricin Protective Antibodies Exhibiting High Affinity from Immunized Non-Human Primates. Toxins.

[41] Cherubin P., Quinones B., Elkahoui S., Yokoyama W., Teter K. (2017). A Cell-Based Fluorescent Assay to Detect the Activity of AB Toxins that Inhibit Protein Synthesis. Methods Mol. Biol..

[42] Damiano J.S., Cress A.E., Hazlehurst L.A., Shtil A.A., Dalton W.S. (1999). Cell adhesion mediated drug resistance (CAM-DR): Role of integrins and resistance to apoptosis in human myeloma cell lines. Blood.

[43] Durand R.E., Sutherland R.M. (1972). Effects of intercellular contact on repair of radiation damage. Exp. Cell Res..

[44] Schuetz J.D., Schuetz E.G. (1993). Extracellular matrix regulation of multidrug resistance in primary monolayer cultures of adult rat hepatocytes. Cell Growth Differ..

[45] Bauwens A., Bielaszewska M., Kemper B., Langehanenberg P., von Bally G., Reichelt R., Mulac D., Humpf H.U., Friedrich A.W., Kim K.S. (2011). Differential cytotoxic actions of Shiga toxin 1 and Shiga toxin 2 on microvascular and macrovascular endothelial cells. Thromb. Haemost..

[46] Mölder A.L., Persson J., El-Schich Z., Czanner S., Gjorloff-Wingren A. (2017). Supervised classification of etoposide-treated in vitro adherent cells based on noninvasive imaging morphology. J. Med. Imaging (Bellingham).

[47] Song K., Mize R.R., Marrero L., Corti M., Kirk J.M., Pincus S.H. (2013). Antibody to ricin a chain hinders intracellular routing of toxin and protects cells even after toxin has been internalized. PLoS ONE.

[48] Rappaz B., Kuttler F., Breton B., Turcatti G. (2015). Digital Holographic Imaging for label-free phenotypic profiling, cytotoxicity, and chloride channels target screening. Methods Pharmacol. Toxicol..

[49] Abraham V.C., Towne D.L., Waring J.F., Warrior U., Burns D.J. (2008). Application of a high-content multiparameter cytotoxicity assay to prioritize compounds based on toxicity potential in humans. J. Biomol. Screen..

[50] Mery B., Guy J.B., Vallard A., Espenel S., Ardail D., Rodriguez-Lafrasse C., Rancoule C., Magne N. (2017). In Vitro Cell Death Determination for Drug Discovery: A Landscape Review of Real Issues. J. Cell Death.

[51] Martin H.L., Adams M., Higgins J., Bond J., Morrison E.E., Bell S.M., Warriner S., Nelson A., Tomlinson D.C. (2014). High-content, high-throughput screening for the identification of cytotoxic compounds based on cell morphology and cell proliferation markers. PLoS ONE.

[52] Bustillo S., Lucero H., Leiva L.C., Acosta O., Kier Joffé E.B., Gorodner J.O. (2009). Cytotoxicity and morphological analysis of cell death induced by Bothrops venoms from the northeast of Argentina. J. Venom. Anim. Toxins incl. Trop. Dis.

[53] Yu H., Chen K., Wu J., Yang Z., Shi L., Barlow L.L., Aronoff D.M., Garey K.W., Savidge T.C., von Rosenvinge E.C. (2015). Identification of toxemia in patients with Clostridium difficile infection. PLoS ONE.

[54] Gal Y., Mazor O., Alcalay R., Seliger N., Aftalion M., Sapoznikov A., Falach R., Kronman C., Sabo T. (2014). Antibody/doxycycline combined therapy for pulmonary ricinosis: Attenuation of inflammation improves survival of ricin-intoxicated mice. Toxicol. Rep..

[55] Sabo T., Gal Y., Elhanany E., Sapoznikov A., Falach R., Mazor O., Kronman C. (2015). Antibody treatment against pulmonary exposure to abrin confers significantly higher levels of protection than treatment against ricin intoxication. Toxicol. Lett..

[56] Otsu N. (1979). A threshold selection method from gray-level histograms. IEEE Trans. Syst. Man Cybern.

[57] Kittler J., Illingworth J. (1986). Minimum error thresholding. Pattern Recognit..

