# Supplementary material for: Whole-Cell Multiparameter Assay for Ricin and Abrin Activity-Based Digital Holographic Microscopy
Source: Toxins (Basel). 2019 Mar 22;11(3):174. doi: 10.3390/toxins11030174 (PMC6468687; doi:10.3390/toxins11030174)
Supplement: Supplementary file 1 [file toxins-11-00174-s001.pdf]

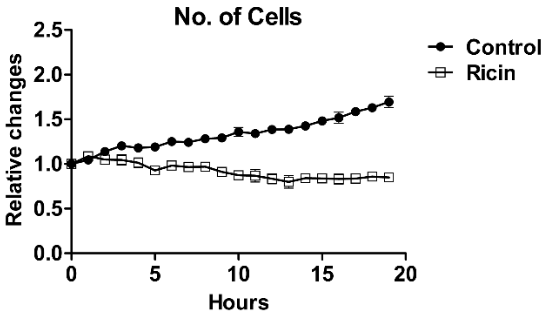

Figure S1. Reduction in cell count by ricin intoxication measured by DHM. HeLa cells were treated with ricin (100 ng/ml) and digital holograms of four different areas in each well were recorded every 10 min for 19 hours. Untreated cells were used as a control. Quantification of the relative change in cell count parameter (mean ±SE) detected using DHM.

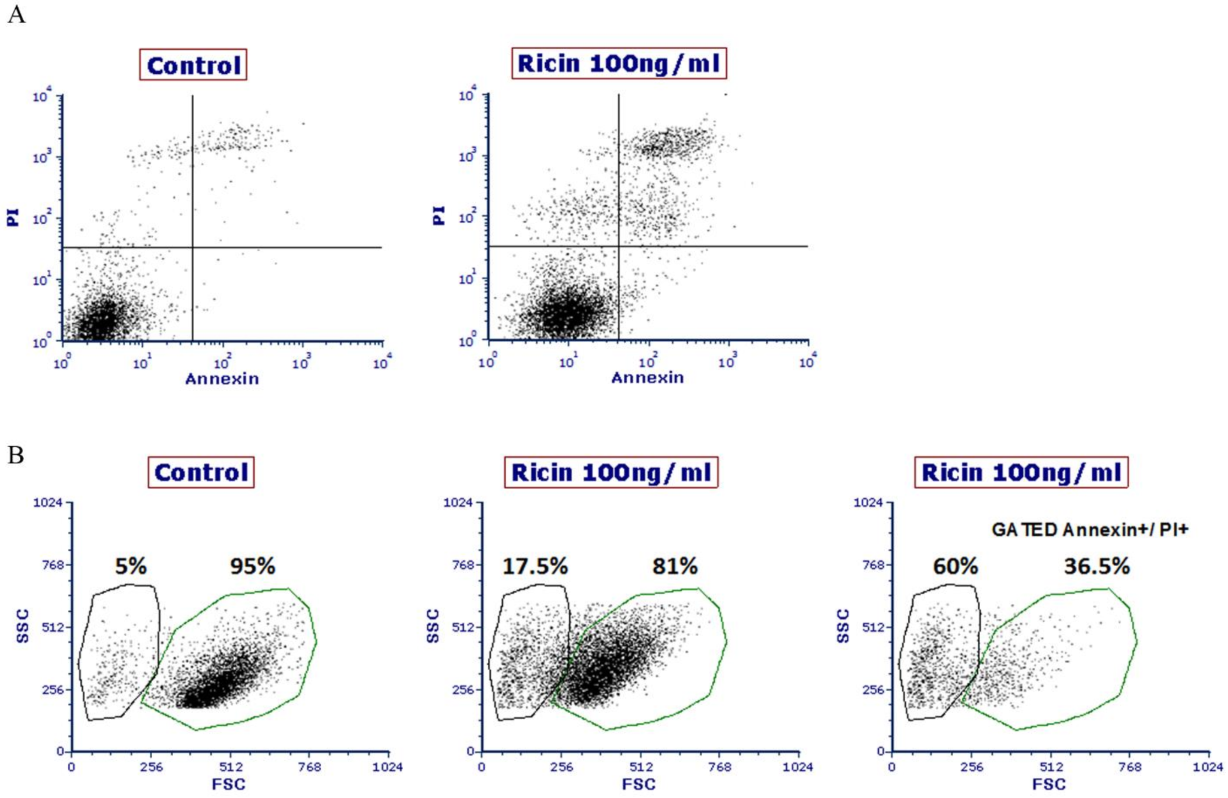

Supplement 2. The induction of cell death by ricin determined by Annexin V/PI apoptosis assay. HeLa cells were exposed to ricin 100 ng/ml for 8 hours (A). A representative FACS analysis of Annexi/PI staining of intoxicated (100

ng/ml) vs. untreated (control) cells. (B) A representative FACS analysis of forward and side scatter (FSC/SSC) parameters of treated and untreated cells. Black gate represents late apoptotic cells, green gate represents live and early apoptotic cells. Left panel- untreated cells as control, middle panel- treated cells, right panel- treated cells gated on annexin+/PI+ double staining population (late apoptotic stage)

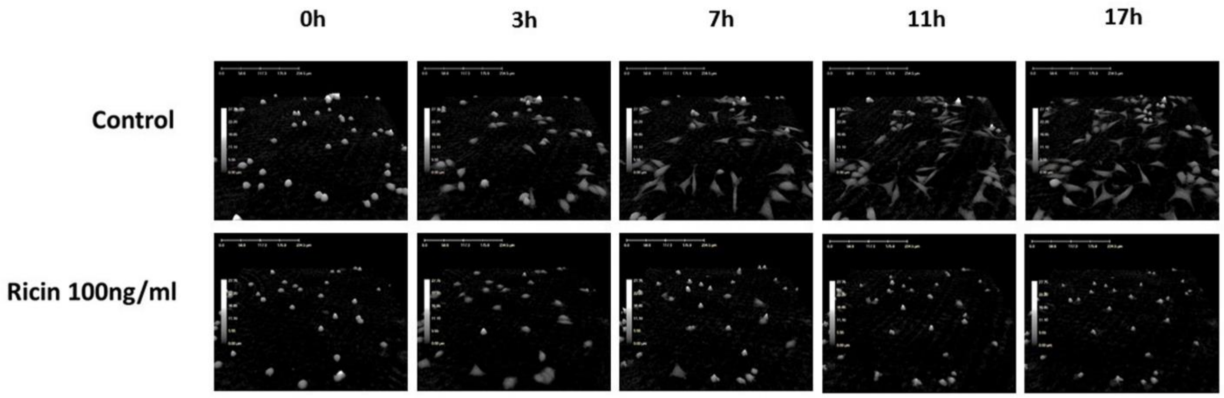

Supplement 3. Morphology features of ricin intoxication of pre-adherent HeLa cells detected by DHM. Effect of ricin treatment on pre-adherent HeLa cells was tested using DHM. Cells were subjected to ricin (100 ng/ml), and digital holograms of four different areas in each well were recorded for 20 hours at 10 min intervals. Untreated pre-adherent cells were used as a control. A representative three-dimensional Images of treated and untreated cells as captured at 0, 3, 7, 11 and 17 hours. Marker for optical thickness depicted on the left side in each image.

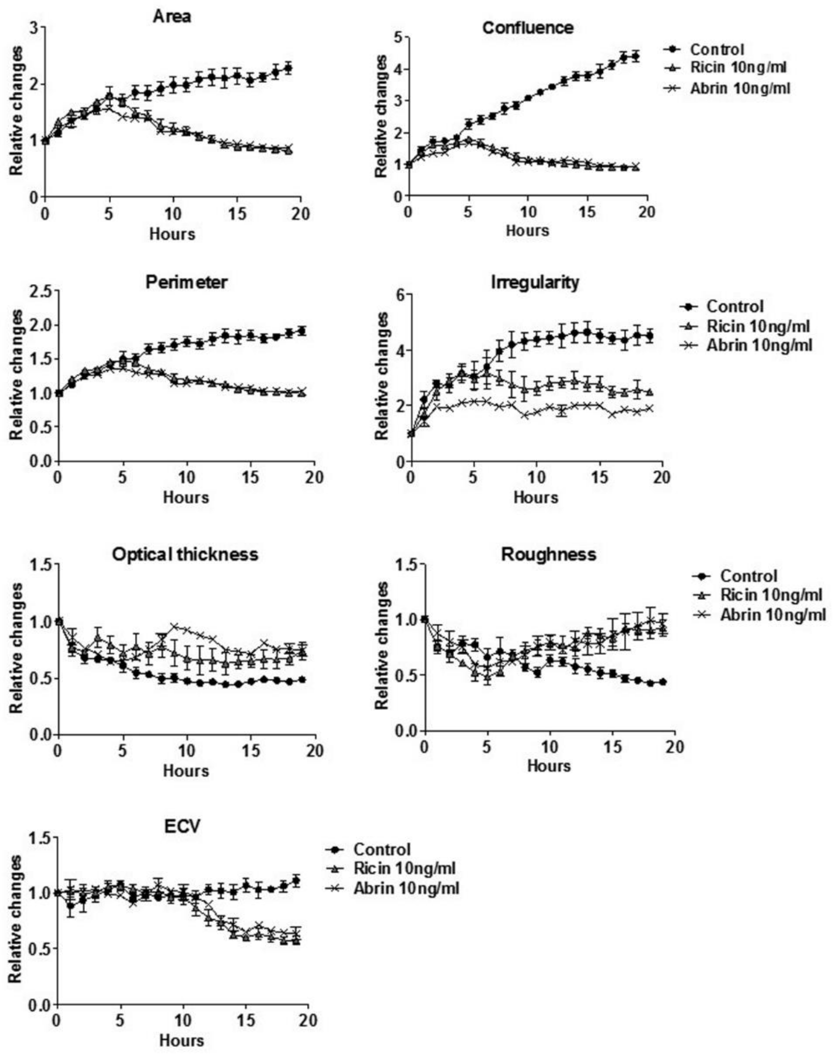

Supplement 4. Similarities in morphology features during RIPs intoxication. Comparison of ricin and abrin intoxication, on various morphology features in HeLa and Vero cell lines. HeLa cells were treated with ricin and abrin (10 ng/ml) and digital holograms of four different areas in each well were recorded every 10 min for 19 hours. Untreated cells were used as a control. Quantification of the relative changes in morphological parameters (mean ±SE) detected using DHM.
